# Sociodemographic determinants of maternal health service use in rural China: a cross-sectional study

**DOI:** 10.1186/s12955-020-01453-6

**Published:** 2020-06-24

**Authors:** Kexin Jiang, Libo Liang, Haifeng Wang, Jingqun Li, Yuze Li, Mingli Jiao, Jingfu Mao, Qunhong Wu

**Affiliations:** 1 Medical department, General Hospital of Heilongjiang Agricultural Reclamation, Harbin, 150088 China; 2 Department of Policy and Hospital Management, School of Public Health, Harbin Medical University, Harbin, 150081 China; 3 Department of ENT, Linkou County Maternal and Child Health Hospital, Mudanjiang, 150081 China; 4 Department of Cardiology, Heihe First People’s Hospital Heihe, Heihe, 157600 China; 5 Harbin NO.6 Senior High School, Harbin, 150000 China; 6 Department of Policy and Hospital Management, School of Health Management, Harbin Medical University, Harbin, 150081 China; 7 Department of Medical Human Resource, School of Public Health, Harbin Medical University, Harbin, 150081 China; 8 Department of Social Medicine, School of Public Health, Harbin Medical University, Harbin, 150086 China

**Keywords:** Maternal health, Health services, Prenatal care, Postnatal care, Policy

## Abstract

**Objective:**

This study examined the relationship between sociodemographic characteristics and maternal health use from a policy perspective. It aimed to provide an overview of maternal health in Heilongjiang’s rural provinces and its implications on rural areas in countries with low and middle income gross domestic products.

**Design, setting, and participants:**

This cross-sectional study used data from the Fifth Health Service Survey of Heilongjiang Province. Participants included 481 mothers who delivered a baby after August 15, 2008. Trained investigators collected data on their family and sociodemographic characteristics, antenatal care, delivery at specialised obstetric institutions (e.g. hospitals, clinics, other medical institutions), and postnatal care services.

**Results:**

The number of women with more than five antenatal care visits and the delivery rate at specialised obstetric institutions were high. Approximately 50% of the participants had three or more postnatal care visits. Maternal healthcare use among women less than 20 years old and those with natural deliveries were higher. There were fewer antenatal care visits among women who had been pregnant once or twice before.

**Conclusions:**

Delivery rates at specialised obstetric institutions and the number of antenatal care visits were higher than the World Health Organization requirements, while the frequency of postnatal care visits were better than most countries. This study identified several demographic characteristics that influenced maternal health service use. Policymakers should consider these findings when developing maternal health policies that protect women’s interests and expand free services. Additional resources should be given to increase the postnatal care capacity and quality of maternal healthcare.

## Introduction

The objective of the World Health Organization’s (WHO) report on maternal healthcare, [1] as stated in Target 5A of the United Nations Millennium Development Goals, was to reduce maternal deaths by 75% between 1990 and 2015. China’s maternal mortality rate was 97 per 100,000 women in 1990 and 27 per 100,000 women in 2015 [2]. Although the number of deaths decreased, the 75% target reduction was not reached. Thus, maternal mortality reduction has become an important goal for the global healthcare system.

In 2015, an estimated 303,000 women died from pregnancy-related causes in China, while 2.7 million babies died within their first 28 days of life, and 2.6 million died from stillbirths [3]. Some studies indicated that increasing maternal healthcare use can efficiently reduce these pregnancy-related deaths [4–6]. Moreover, factors such as antenatal care (ANC), delivery at a specialised obstetric institutions (e.g., hospitals, clinics, and other medical institutions), and postnatal care (PNC) services serve as key health interventions that reduce maternal and new-born morbidity and mortality [7].

The WHO recommended promoting ANC to have a positive pregnancy experience, and increasing the recommended number of ANC visits from four to eight by 2016 [3]. However, it reported that only 64% of women had ≥4 ANC visits worldwide. The recommendations for visits vary between countries: 7 in France, [8] 13 in the US, and 5 in China [9]. Moreover, since the maternal mortality rate is only 10 in 10 million [10] in countries where births occur in a hospital, the WHO recommended that pregnant women should give birth in hospitals. Additionally, PNC services should be included. Research [11] has indicated that 91% of obstetric haemorrhage deaths and all deaths from pregnancy-induced hypertension occurred within 7 days after delivery, while 78% of deaths due to infection occurred two to 8 days after delivery. Therefore, timely and effective PNC and early abnormalities detection can prevent maternal deaths.

In 2015, approximately 830 women died every day due to pregnancy and childbirth complications worldwide, with over 95% of those deaths occurring in sub-Saharan Africa and Asia [12]. The WHO stated that health expenditure per capita was less than $45 a year for those without basic health care access [13], but higher healthcare costs do not necessarily improve health outcomes. For example, the average world per capita health expenditure is US $1058. In 2014, the United States’ average per capita health expenditure was US $9402, which is twice the average for other developed countries. Despite this, the US maternal mortality rate was still 14 per 100,000 which is above average for most developed countries. By contrast, Sri Lanka’s [14] average per capita health expenditure was US $127, but owing to several government initiatives, its current maternal and infant mortality rates are now significantly lower than other countries at the same economic level. Similarly, despite China’s per capita health expenditure of US $419, China’s primary healthcare system [15], including maternal care, has achieved remarkable results.

Research has attributed China’s performance to traditional influential factors like household wealth [16], distance [17], education [18], and other major factors [19, 20]. However, recent studies suggested that national policies directly reduce the effects of these individual and social factors [21, 22]. For example, between 1978 and 2015, policies gradually evolved and applied differently to certain groups. The one-child policy in 1978 was changed to the two-child policy in 2015. Despite this, before 2015, rural or pregnant women who had a girl first could still have a second child. Thus, understanding the impact of China’s policies on maternal health services is essential in improving maternal health outcomes.

The WHO recommended that community agencies should provide essential maternal health services [23]. According to the China Yearbook 2017 [2], with a national population of 1.379 billion, the number of all medical institutions in China was 983,000. Of these, 29,140 were professional hospitals fully capable of providing maternal services over long distances. The primary healthcare system in China [15] includes the rural maternal service, a policy designed to provide fair and accessible services for high and low income women regardless of education level. Access to five ANC visits and hospital delivery are equally guaranteed and used by all rural women in China [21] at no cost. In recent years, China’s primary care health service expanded its capacity [24], and the minimum national basic public health expenditure standard was raised from 15 Yuan ($2.20) per person in 2009 to 50 Yuan ($7.41) per person in 2017 [25]. Such is important for realising fair use in health services, improving service use, and reducing deaths and complications.

Women from lower socioeconomic backgrounds may be at risk for lower healthcare use levels [26]. China has a vast geographic area, and while the policies are uniform, economic development is imbalanced. Heilongjiang, the central region of China, has obvious economic and environmental differences compared to the eastern and western regions. The rural area of Heilongjiang is particularly representative. Thus, this study provides an overview of women’s health service use in Heilongjiang’s rural areas and reports on observed changes in use based on the National Health Service Survey (NHSS) data. We examined factors influencing the likelihood of pregnant women having 5+ ANC and 3+ PNC visits.

## Methods

### Sample location

We conducted the study in a rural area in Heilongjiang Province, northeast China, with 16,654,000 people (43.5% of the province’s population). The majority of the county’s population was engaged in farming [27]. According to the national Gross Domestic Product (GDP) rankings, this county was representative of less developed rural areas in China [28, 29]. Moreover, the last national census provided that the fertility rate in Heilongjiang was 1.03%, which was below the national level of 1.5%. In terms of international standards, less than 1.3% is considered an ‘ultra-ultra-low birth rate’ [30]. The maternal mortality rate in Heilongjiang’s rural areas was 18.4 per 100,000, which was lower than the average national mortality rate of 19.6 per 100,000 in China.

### Data

The data were from the fifth Health Service Survey of Heilongjiang Province which was part of China’s Fifth NHSS in 2013. The NHSS is a nationally representative survey commissioned by the National Health and Family Planning Commission of China (NHFPC), with details regarding its sampling method and quality assurance measures appearing in other papers [31–33]. The survey is conducted every 5 years; at the time of writing, the latest available survey data were for 2013, with the 2018 survey in progress.

We used a four-stage stratified random sampling scheme to ensure a representative sample of Heilongjiang Province’s population. First, we chose nine counties from which 35 townships were selected, then from these townships 78 communities were selected, and finally, from these 78 communities 5289 households were selected.

**Fig. 1:**
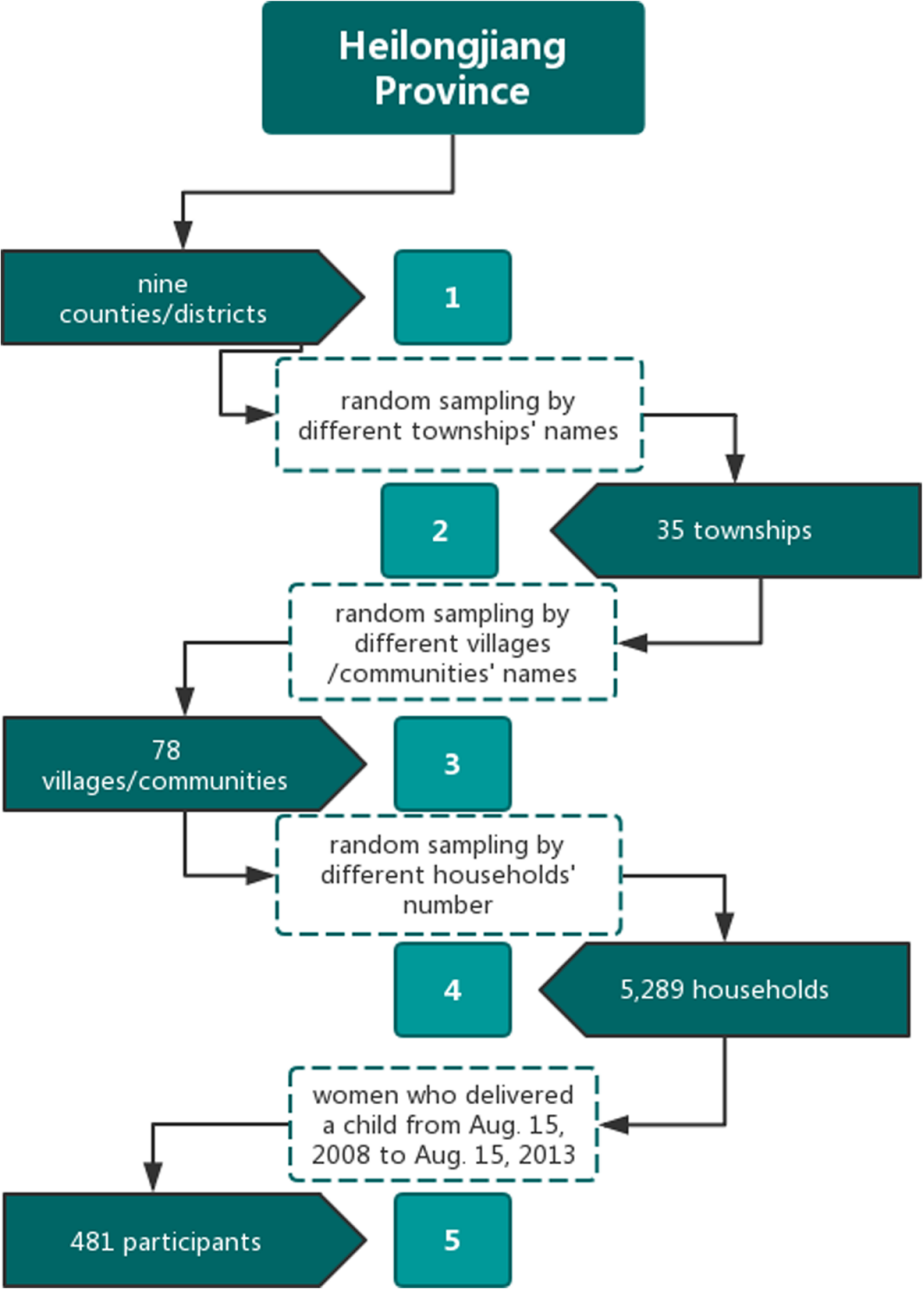
Flow chart of random sampling based on this study

In this study, we examined women who delivered a child between August 15, 2008 and August 15, 2013. We excluded respondents living in urban areas from the analysis leaving 481 women as participants (Fig. 1). The structured questionnaire survey was conducted by the NHFPC. All participants completed face-to-face interviews with professionally trained investigators about their family and sociodemographic characteristics, insurance, ethnicity, education, age at the time of last childbirth, child’s sex, delivery method, pregnancy number, and delivery number. There was a special focus on maternal healthcare, including a number of ANC and PNC visits, and delivery location.

### Study variables

Delivery at a specialised obstetric institution was defined as giving birth in a township hospital or higher-level healthcare facility, which has obstetric medical professionals, dedicated delivery rooms, and emergency obstetric care, as opposed to a village clinic or home. The maternal healthcare management requirement guidelines in China suggest that pregnant women must have at least five ANC and three PNC visits [9, 34]. This study used three main indicators to reflect maternal healthcare services use: whether the participant (1) had at least five ANC visits; (2) had her delivery at a specialised obstetric institution; and (3) had at least three PNC visits.

We examined different demographic characteristics which includes the proportion of people using maternal health services, the number of ANC and PNC visits, delivery at specialised obstetric institutions, and health insurance status so that pregnant women could receive compensation for medical services. There are 55 ethnic groups in China with Han being the largest; thus, we added “ethnicity” as a research variable. Finally, we divided education into three levels: **≤** junior high school was defined as primary; college undergraduate degree as secondary; and **≥** master’s degree as post-secondary.

### Statistical analysis

We used descriptive statistics and chi-square analyses for three indicators: ANC use, delivery at a specialised obstetric institution, and PNC services use. We assessed the predictors of ANC and PNC visits with reference to the most recent birth. Through the variable selection process, we entered the variables that met the requirements into a multiple logistic regression model to analyse the factors influencing ANC and PNC visits. Odds ratios (OR) and 95% confidence intervals (CI) were calculated, and *p* < 0.05 was considered statistically significant. All statistical analyses were performed using the IBM software SPSS Statistics V. 21.0.

### Ethics approval

The study protocol was reviewed and approved by the research ethics committee of Harbin Medical University. In the NHSS, the Chinese National Bureau of Statistics institutional review board provided survey review and ethics approval. All respondents read a statement explaining the purpose of the survey and gave their informed written consent to participate.

## Results

The study showed that 0.2% of pregnant women in Heilongjiang Province’s rural areas did not attend any ANC visits, 2.3% delivered in non-specialised obstetric institutions, and 3.5% did not have a PNC visit within 42 days post-delivery. Specific indicators reached 85.9% for 5+ ANC visits and 46.9% for 3+ PNC visits.

As shown in Table 1, 97.3% of women in rural areas of Heilongjiang Province chose professional institutions for delivery. Therefore, these analyses focused on factors affecting ANC and PNC visits. For variable age at the time of last childbirth, 96.7% of women under 20 attended ANC visits and 56.7% attended PNC visits, while women over 35 had percentages of 79.4 and 41.3% respectively. In terms of delivery method, the ANC and PNC rates of pregnant women with natural delivery were higher than those who delivered by caesarean section. For child’s sex, the compliance rates for boys were 92.4 and 49% while maternal healthcare use was lower for girls. These differences were all statistically significant.

**Table 1:** Variations in indicators of maternal services use

Factors	N	Percent reporting use of:		
		ANC Visits 5+	Delivery at specialised obstetric institutions	PNC Visits 3+
**Health Insurance**				
**No**	14	78.6	100.0	35.7
**Yes**	467	86.1	97.2	47.2
**Ethnicity**				
**Non-Han**	13	92.3	92.3	38.5
**Han**	468	85.7	97.4	47.1
**Education**				
**Primary**	90	84.4*	98.9*	48.9
**Secondary**	370	86.2	97.0	46.1
**Post-secondary**	21	85.7	95.2	52.4
**Age at time of last childbirth**				
**Under 20**	30	96.7*	100.0	56.7*
**20–24**	193	85.5	98.4	47.7
**25–29**	107	87.9	96.3	48.6
**30–34**	88	85.2	95.5	43.7
**Above 35**	63	79.4	96.8	41.3
**Child’s sex**				
**Female**	231	87.9	97.4	44.6
**Male**	250	92.4	97.2	49.0
**Delivery method**				
**Natural childbirth**	293	90.8*	95.9**	53.2**
**Caesarean section**	188	89.4	99.5	36.9
**Number of pregnancies**				
**1**	250	85.2*	97.6*	47.6
**2**	169	84.0	95.9	42.3
**3+**	62	93.5	100.0	56.5
**Number of deliveries**				
**1**	304	86.2*	98.0*	48.0
**2+**	177	85.3	96.0	44.9
**All respondents**	481	85.9	97.3	46.9

Note: *p < 0.05; **p < 0.01

Table 2 shows the full model results that evaluated the influence of all factors. ANC visits among pregnant women under 20 years old were significantly higher (OR = 0.08) compared with those who had caesarean sections. Women who had natural delivery have higher odds of attending 3+ ANC visits. For pregnant women who had previously been pregnant once or twice, ANC visits were lower than those pregnant three times or more. For PNC visits, delivery method had a significant effect; pregnant women who choose natural childbirth (OR = 0.50) had higher odds of having more PNC visits compared to women with caesarean sections. Other variables did not show any significant differences.

**Table 2:** Results of the multilevel analysis of indicators related to maternal health services use

Characteristics	Odds Ratio (95%C I)	
	ANC visit 5+	PNC visit 3+
**Health Insurance (ref: Yes)**		
**No**	2.01 (0.52–7.76)	1.75 (0.57–5.39)
**Ethnicity (ref: Han)**		
**Non-Han**	0.36 (0.04–2.88)	1.12 (0.35–3.62)
**Education (ref: Post-secondary)**		
**Primary**	1.16 (0.29–4.60)	1.17 (0.44–3.11)
**Secondary**	0.97 (0.27–3.52)	1.24 (0.50–3.08)
**Age at last birth (ref: above 35)**		
**Under 20**	0.08* (0.01–0.69)	0.39 (0.14–1.08)
**20–24**	0.41 (0.16–1.07)	0.62 (0.30–1.29)
**25–29**	0.42 (0.16–1.06)	0.66 (0.33–1.30)
**30–34**	0.597 (0.25–1.44)	0.84 (0.43–1.66)
**Child’s sex (ref: Male)**		
**Female**	1.25 (0.74–2.13)	1.18(0.81–1.72)
**Delivery method (ref: Caesarean section)**		
**Natural childbirth**	0.53* (0.31–0.90)	0.50*(0.34—0.73)
**Pregnant number (ref:** 1)		
**1**	4.25* (1.05–17.29)	1.67 (0.78–3.58)
**2**	3.17* (1.03–9.73)	1.81 (0.99–3.33)
**Delivery number (ref:** 2**+)**		
**1**	0.93 (0.30–2.93)	1.07 (0.52–2.23)

Note: *p < 0.05

## Discussion

This study found that the overall percentage of deliveries at professional institutions (97.3%) was higher than those with 5+ ANC visits (85.9%), while both were significantly higher than the 3+ PNC visits (46.9%). These findings were higher than those found in other developing countries such as Nigeria (ANC 60.3%, professional institution delivery 43.4%, and PNC 41.2%) [20], Liberia (ANC 42.5%, professional institution delivery 49.7%, and PNC 36.0%) [17], and India (ANC 61.7%, professional institution delivery 49.8%, and PNC 37.4%) [35].

ANC service use and hospital delivery, both common in China, are essential in preventing and reducing neonatal birth defects [1]. Our study shows that numerous factors affect ANC use, including age at last childbirth, delivery method, pregnancy number, and delivery number. Women under 20 years old had increased odds of attending 5+ ANC visits as compared with older women, which is consistent with previous researches [36, 37]. This may be because younger mothers highly value their pregnancy, or because maternal health awareness has gradually improved with the continuous improvement of national quality and education level. Furthermore, women who delivered naturally had a greater likelihood of ANC visits compared to women with caesarean sections [38]. On the one hand, examination frequency can predict a pregnant women’s medical compliance. On the other hand, diversified prenatal education such as parenting and birthing classes greatly increase the likelihood of a natural birth. Those who did not use ANC visits were more likely to give birth by caesarean section. Therefore, women may be motivated to complete more ANC visits [39].

The present findings are consistent with previous analyses [40, 41] which state that socioeconomic determinants correlated with ANC visits. One intriguing result of our study was that as the number of pregnancies increased, maternal healthcare use in the form of ANC visits also increased [42]. A possible reason for this finding is that women with 3+ pregnancies gave more importance to their ANC visits because they were worried about the antenatal check-up process. Moreover, the rate of single-child mothers in Heilongjiang Province’s rural areas (63.2%) was far higher than the national average [2]. However, the policy on free ANC visits was open only to first-time pregnancies, while women who became pregnant multiple times were ineligible to receive subsidies [8]. Therefore, we recommend that all pregnant women are provided with the same healthcare access.

Our study showed PNC visits were mainly affected by delivery method, with caesarean deliveries resulting in significantly lower PNC visits than natural births. In developing countries, the ANC visit use is generally high while PNC visit use is low. Our studies show that PNC visits are uncommon in China. Tao et al. identified several factors which influence the lack of PNC provision and use [43]. They included inadequate government funding, insufficient number of professional PNC staff, providers’ focus on income-generating services, and women’s lack of awareness about PNC benefits. Additionally, more attention was given to the new baby’s health, reducing the attention on maternal health post-delivery. Therefore, increasing health education [44, 45] about PNC benefits is important. Furthermore, the assessment index [46] is comprehensively utilised in provinces while the country is spot-checked; thus, the quality of maternal health across China cannot be guaranteed. Specifically, there is a lack of quality assessment indicators for PNC visits.

Since 2000, the National Health and Family Planning Commission of China issued a series of legislation to improve maternal health [41] through public health service project subsidies, including the New Rural Cooperative Medical Scheme (NCMS) insurance, major illness insurance, and medical assistance. Specifically, these policies aimed to reduce ANC and hospital delivery costs in rural areas and increase maternal prenatal screening use and hospital delivery rate. The general goal was to establish a free maternal health examination system throughout the country so that every pregnant couple can enjoy free maternal health examination services, reducing birth defect risks and improving the health of the birth population. These policies promote equity in maternal health services use between urban-rural areas and provinces. Government policies [47] may influence higher delivery rates at professional organizations since hospital deliveries are covered by the NCMS, and there is a policy restricting birth certificates and healthcare cards to children born in specialised obstetric institution so that they may receive government support.

### Limitations

There were several limitations to this study. First, the survey and quantitative data collection tools were designed to investigate maternal services use during pregnancy only, and there was a lack of qualitative research to analyse maternal needs. Second, the data were provided through self-report which can lead to recall bias. However, the data were collected within 5 years of childbirth. Since pregnancy and childbirth are major life events, the recall bias is assumed to be small. Third, although our sample was designed to broadly reflect Heilongjiang Province’s rural areas, we did not obtain a representative sample of rural China. Fourth, since the national survey cannot expand the final sample size, its representativeness may be insufficient. Nevertheless, the results provide a preliminary overview of Chinese maternal health services use.

## Conclusions

The rates of using at least five ANC visits and delivery at specialised obstetric institution were higher in our sample than the level recommended by the WHO [48]. This trend could be the result of government programs that recognize ANC benefits in improving health outcomes for women and children. However, there are still many challenges reflected by low PNC use rates [42], such as inadequate funding, lack of skilled human resources, and unawareness of PNC benefits. The WHO recommendations should be followed, namely, adding key maternal care indicators [1] related to interventions received during ANC visits, normal childbirth care, and PNC visits into the national household health surveys. Therefore, population-based estimates of these indices would be available. At the same time, future research should include both qualitative and intervention designs.

Our results suggested that demographic characteristics influence women’s maternal health services use. Interventions promoting maternal health services might consider these findings to increase their effectiveness. Protecting the rights and interests of pregnant mothers with multiple pregnancies and expanding the provision of no-cost maternity services are vital. The findings also suggest that expanding the available PNC visit resources would strengthen the capacity to offer comprehensive maternal healthcare services. The results provide an overview of healthcare use in Heilongjiang’s rural area as well as suggestions for maternal health services in rural areas in low and middle income GDP countries.

## Data Availability

Datasets used or analysed during the current study are available from the corresponding author on reasonable request.
